# vitisFlower^®^: Development and Testing of a Novel Android-Smartphone Application for Assessing the Number of Grapevine Flowers per Inflorescence Using Artificial Vision Techniques

**DOI:** 10.3390/s150921204

**Published:** 2015-08-28

**Authors:** Arturo Aquino, Borja Millan, Daniel Gaston, María-Paz Diago, Javier Tardaguila

**Affiliations:** Instituto de Ciencias de la Vid y del Vino (University of La Rioja, CSIC, Gobierno de La Rioja), 26006 Logroño, Spain; E-Mails: arturo.aquino@unirioja.es (A.A.); borja.millanp@unirioja.es (B.M.); daniel.gaston@unirioja.es (D.G.); maria-paz.diago@unirioja.es (M.-P.D.)

**Keywords:** Android application, OpenCV4Android, OpenCV library, image analysis, grapevine flower counting, yield prediction, *Vitis vinifera* L., precision viticulture, precision agriculture

## Abstract

Grapevine flowering and fruit set greatly determine crop yield. This paper presents a new smartphone application for automatically counting, non-invasively and directly in the vineyard, the flower number in grapevine inflorescence photos by implementing artificial vision techniques. The application, called vitisFlower^®^, firstly guides the user to appropriately take an inflorescence photo using the smartphone’s camera. Then, by means of image analysis, the flowers in the image are detected and counted. vitisFlower^®^ has been developed for Android devices and uses the OpenCV libraries to maximize computational efficiency. The application was tested on 140 inflorescence images of 11 grapevine varieties taken with two different devices. On average, more than 84% of flowers in the captures were found, with a precision exceeding 94%. Additionally, the application’s efficiency on four different devices covering a wide range of the market’s spectrum was also studied. The results of this benchmarking study showed significant differences among devices, although indicating that the application is efficiently usable even with low-range devices. vitisFlower is one of the first applications for viticulture that is currently freely available on Google Play.

## 1. Introduction

Precision agriculture proposes the development and use of new technologies for improving crop management and quality. In the field of viticulture, there has been an increasing interest over the last few years in the development of innovative image-based techniques for objective vineyard monitoring [1,2,3,4,5,6]. This approach would allow one to increase management efficiency by providing more accurate control of agronomic parameters. Undoubtedly, it would produce an outstanding positive impact on grape-growing sustainability, as well as on grape and wine quality.

Flowering and fruit set (rate of flowers becoming grapes) are two physiological processes that strongly determine grapevine yield [7]. Furthermore, 30% of yield seasonal variation is associated with the number of berries per cluster and 60% with the number of clusters per vine [8,9], impacted by the pruning load and bud fruitfulness. The knowledge of the rate of fruit set at very early stages (prior to bunch closure) is of great value for grape growers, as this variable can be used to estimate or predict the final yield at harvest, provided a historical value of average berry weight, and the average cluster number per vine for each vineyard is available for each vineyard. Flowering and fruit set, together with berry size, have also a great impact on grape and wine quality, since they define the number of berries per cluster and contribute to determining the cluster architecture and compactness, which are a recognized key indicators of grape and wine quality [10]. Due to their importance and multi-factorial variability [7,11,12], there are a great number of viticultural actions aimed at controlling their behavior [7,13,14,15,16,17,18,19]. So far, flowering and fruit set cannot be accurately assessed, since manual flower counting is unfeasible, as it is extremely time and labor demanding, besides being mostly destructive.

The huge recent progress of mobile devices (also known as smartphones) has opened a wide range of opportunities that were previously unviable. Their portability, accessibility, computing performance and the high quality of the cameras they currently include are features that have enabled the development of innovative applications in fields, like medicine, sport, geography and agriculture, among others. Specifically, in viticulture, there are still not many examples of smartphone applications. One of them was recently presented by De Bei *et al*. [20,21]. These authors developed an application for measuring grapevine canopy architecture using image analysis techniques on images acquired with the device’s camera. The application was developed exclusively for iOS smartphones, not being commercial yet.

The goal of the present work was to develop and to test the reliability and computational efficiency of a novel smartphone application, called vitisFlower^®^, for automatically, efficiently and non-invasively counting flowers in grapevine inflorescence images taken directly in the vineyard. This application benefits from the fact that the number of flowers in an inflorescence image is strongly correlated to the actual flower number in the real inflorescence [22], to provide the user with a powerful tool for flowering assessment. The application, called vitisFlower^®^, was developed and implemented for Android devices with the aim of maximizing its availability to users, since this operating system is the most extended worldwide [23]. The application was tested following a double approach. On the one hand, the application was tested by taking and analyzing 140 inflorescence images of 11 grapevine varieties using two different devices: a high-end and a mid-range device. In this way, not only its accuracy in detecting grapevine flowers was evaluated, but also its reliability to properly work on devices of different capabilities. On the second hand, the application’s computational efficiency was also evaluated by performing a benchmarking study using four devices covering the whole market spectrum.

## 2. Experimental Section

### 2.1. Image Analysis for Flower Counting in Grapevine Inflorescences

vitisFlower^®^ is a newly-developed application for Android devices that allows one to take a photo of a grapevine inflorescence for its analysis. This analysis, based on the methodology proposed and validated in [22], implements artificial vision algorithms aimed at counting the number of flowers per inflorescence in the image.

The methodology for counting grapevine flowers is based on mathematical morphology and statistical techniques. It has a pre-requisite, which involves taking the photo by placing dark cardboard behind the inflorescence for allowing its segmentation from the background. Once the image is correctly acquired, the methodology can be divided into three steps:

- Image pre-processing: this step basically consists of automatically segmenting the inflorescence from the background using color discrimination criteria (invariant to light conditions) for computing a region of interest (ROI).
- Image analysis: in this step, the detection of flower candidates is achieved. Flowers are quasi-spherical in shape, so they produce a point of maximum light reflection. Therefore, flower candidates are identified in the area of the image delimited by the ROI as those connected components being regional maxima in the lightness channel of the Lab color space (concretely the Lab space used was CIE 1976 L*a*b* [24]).
- Image post-processing: this final stage intends to remove those regional maxima not corresponding to real flowers in the image. It is carried out by sequentially applying these two statistical filters:
  1. Region size filter: removal of those candidates with a size larger than expected, taking into account the statistical size distribution of the candidates.
  2. Shape filter: due to the geometry of a flower, the area of maximum light reflection on its surface is expected to describe a quasi-circular shape; therefore, this filter eliminates those candidates describing elongated configurations.

**Figure 1:**
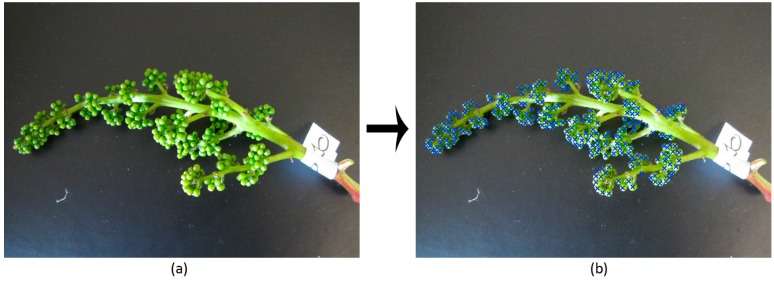
(**a**) Grapevine photo taken in the vineyard using black cardboard as the background; (**b**) result of the image analysis algorithm overviewed in Section 2.1 for automatically detecting flowers. Detected flower centers are marked with blue crosses.

Once flower candidates are filtered, the remaining ones are definitely considered as real flowers and counted. As an example, Figure 1a shows a photo of a grapevine inflorescence on dark cardboard, whereas Figure 1b illustrates the result of its analysis by representing detected flowers with blue crosses.

### 2.2. Application’s Technical Overview

The vitisFlower^®^ application integrates the image analysis methodology previously described with the image capture process and the results storage to provide vine growers with a unique and comprehensive tool for counting grapevine flowers in the vineyard. For its implementation, technical and design decisions were taken for finding the best trade-off between accessibility, efficiency and portability.

With the aim of maximizing the availability of the application to potential users, the first technical decision was its development for Android-powered smartphones and tablets, since this operating system is the dominant one in the mobile-device market worldwide [23]. In addition, this accessibility was also favored by guaranteeing the compatibility of its implementation with Android Version 2.3 and above.

As discussed, the selection of Android as the operating system provides outstanding accessibility advantages. On the contrary, it also implies certain limitations in terms of efficiency and versatility for the needs of this work, especially for the implementation of the image analysis algorithms. In this respect, OpenCV is a highly optimized library, written in C/C++, that offers a wide catalogue of image processing and analysis functions. Android applications are mainly developed in Java, with an existing Android version of this library called OpenCV4Android. However, the original C/C++ version was used instead of its Android version to implement the core of the image analysis methodology due to the following reasons:

- The use of OpenCV4Android through Java code introduces limitations in image handling, which influence efficiency. In this respect, C/C++ provides higher versatility than Java, allowing image handling at lower levels.
- The power and versatility that is offered by C/C++ pointers allows one to reduce memory usage by reducing image and other variable copies. This advantage considerably helped to keep the application within the standard Android heap size.
- The implementation of the core using the standard C/C++ OpenCV library instead of its Android version outstandingly increases its portability, since there are OpenCV compilations for numerous operating systems, like iOS and Windows, among others.
- The use of the standard C/C++ OpenCV for implementing the image analysis algorithms facilitates their development and testing, since it can be carried out directly on a PC, avoiding the need for the use of smartphones or emulators to this effect.

**Figure 2:**
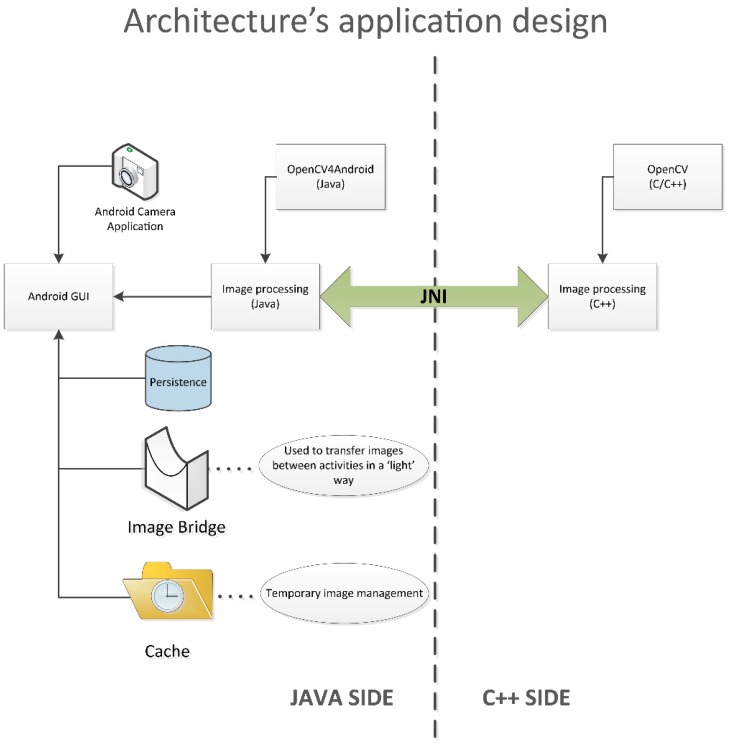
Architecture design of the vitisFlower^®^ Android application.

The inclusion of the C/C++ OpenCV library for implementing the core of image analysis algorithms highly influenced the application’s architecture design, as can be seen in Figure 2.

The application’s architecture was basically composed of two blocks, one implemented in Java and the other one in C++. The “C++ side” implements the algorithm described in Section 2.1 using the OpenCV library, offering a ready-to-use compiled library. On the other hand, the “Java side” implements the whole application with the exception of the “*ad hoc*” developed functions. Hence, it implements the main application’s body and the graphical user interface; it is in charge of coordinating tasks; it calls the Android camera application for taking an image; it is responsible for appropriately storing the results, *etc.* When an image is captured on the “Java side” and its analysis is required, this is requests the “C++ side”. Nevertheless, the direct communication between both blocks was unfeasible, and the use of the Java Native Interface (JNI) for allowing this interaction was required. This way, the “Java side” invokes the desired functionality through JNI, which is really in charge of executing the C++ library and returning the results.

### 2.3. Application’s Performance Description

vitisFlower^®^ was designed to be used by all kinds of smartphone users. It was achieved by means of the following two decisions:

- Implementation of a simple and friendly graphical user interface: the interface shows only the relevant information to the user, preferably by using symbols or illustrations instead of descriptive text.
- Linear execution: the application has a user-independent execution line in which the user is exempted from making any important decision. It allows the user to utilize the application without any knowledge about its internal performance.

**Figure 3:**
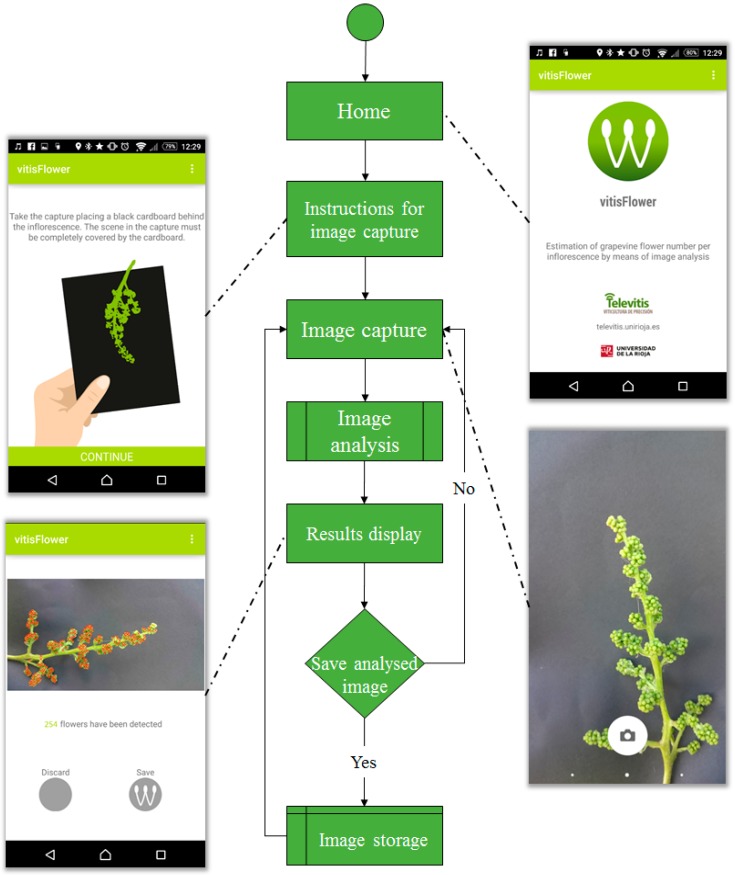
Flow-chart diagram of the vitisFlower^®^ application illustrated with the application’s screenshots.

Figure 3 shows an illustrated flow-chart diagram of the application, in which the different stages can be described as:

1. Home: the application shows the vitisFlower^®^ logo along with basic information about the aims and authorship.
2. Instructions for image capture: the application briefly informs the user of some basic notions for appropriately taking a photo.
3. Image capture: the camera application available in the user’s Android device is invoked to make a capture. If the camera application is properly configured, it shows the captured image and allows one to discard it to take a new one in case the previous one was not properly acquired, for example because of the presence of leaves in the image, or due to the fact that the scene was not correctly focused, or it was overexposed.
4. Image analysis: this state is transparent to the user. It is in charge of analyzing the image taken in the previous state for detecting and counting flowers. For reducing the computational workload, the image is scaled down to a resolution of 2 Mpx prior to its analysis.
5. Results display: the results of the image analysis are presented to the user. On the one hand, the image with the detected flowers marked with red crosses is displayed. It easily allows one to graphically inspect the obtained results. On the other hand, the number of detected flowers is also shown. At this point, the user decides to save the results or to discard them if they are not satisfactory.
6. Image storage: this stage is reached if the user decided to save the results in the previous step. The processed image is saved in a folder called “VitisFlowerImages” created by the application and located in the root folder of the device’s internal storage. The image is saved and named as follows [name]_[date]_[detected number of flowers].jpg, where:
  - [name]: a dialog box allowing one to insert an image name. If it is omitted, this field takes the value “image”.
  - [date]: the complete date of the image capture with the following format and information: day-month-year_hour.minutes.seconds.miliseconds.
  - [detected number of flowers]: the number of flowers detected in the image.

Additionally, the user can read the complete information about the application’s copyright and technical issues by clicking the icon composed of three white dots located in the upper-right corner of the window (see any application’s screenshot in Figure 3 to identify this icon).

### 2.4. Testing and Validation of the Application

#### 2.4.1. Application’s Performance Evaluation

The application’s performance was evaluated with a double purpose: the assessment of its ability to accurately detect flowers and the comparison of its performance using the smartphone’s cameras. To achieve these goals, an experiment using 2 devices of different capabilities was designed. The vitisFlower^®^ application was installed on a high-end and a mid-range device; the Sony Xperia Z2 (Sony Corp., Tokyo, Japan) and BQ Aquaris E5 (Mundo Reader S.L., Madrid, Spain), respectively (see Table 1 for the features of both smartphones relevant to this experiment). These devices were separately used to acquire and analyze 70 images of 7 different grapevine varieties (10 images per variety), producing a total of 140 analyzed images. The considered varieties were: Airen, Cabernet Sauvignon, Chardonnay, Grenache, Riesling, Syrah, Tempranillo, Merlot, Chenin Blanc, Sauvignon Blanc and Semillon. The images were taken at pre-flowering stage denoted as BBCH 55, according to the scale of Lorenz *et al.* [25], in a grapevine variety collection located in the experimental vineyards of the “Instituto de Ciencias de la Vid y el Vino” (Logroño, Spain). Then, the produced outcomes were evaluated using the following metrics based on contingency tables for binary classification:

(1) $$RC=\frac{TP}{TP+FN};PC=\frac{TP}{TP+FP}$$

**Table 1:** Main relevant features of the 2 devices used for evaluating the performance of the vitisFlower application.

	Feature	Price/Release Date	Sensor Model	Resolution	Lens Size	Aperture	ISO
Device							
**Sony Xperia Z2**		549.0 €/2014	Sony IMX220	20.7 Mpx	1/2.3″	f/2.0	50–800
**BQ Aquaris E5**		209.90 €/2014	Sony IMX214	13 Mpx	1/3.2″	f/2.2	100–1600

Metric *RC* denotes *Recall*, which provides the percentage of actual flowers detected by the algorithm, whereas *PC* stands for *Precision*, which calculates the percentage of flowers correctly detected. For allowing the calculation of these metrics, a gold standard set was created. It was performed by manually labelling flowers on each of the 140 images acquired with both smartphones, making use of a PC software specifically developed in MATLAB (MatlabR2010b, MathWorks, Natick, MA, USA) to this effect. Thus, true positives (*TP*), false positives (*FP*) and false negatives (*FN*) were calculated and annotated per image as follows:

- *TP*: the number of flowers automatically detected corresponding to the actual flowers labelled in the gold standard.
- *FP*: the number of flowers automatically detected that do not correspond to actual flowers in the gold standard. Redundant *TP*s (a redundant true positive is when a flower is detected more than once) were also considered as *FPs*.
- *FN*: the number of actual flowers labelled in the gold standard that were not automatically found.

#### 2.4.2. Application’s Computational Efficiency Study

The usability of the application is highly influenced by the time it takes to analyze an image. Moreover, its accessibility is strengthened if this computation time does not exceed reasonably values for a wide range of smartphones. Therefore, to evaluate these attributes, the application was tested from a computational point of view. This study basically consisted of studying the computation time consumed by 4 smartphones with different hardware and software configurations for analyzing the same set of images. The devices were selected to cover a wide range of the market’s spectrum in terms of price and performance; the selected ones were: the Sony Xperia Z2, Sony Xperia Z2 Tablet, BQ Aquaris E5 and Motorola Moto G (2013 version) (Motorola Mobility, IL, USA). Table 2 shows the features relevant for this study of the 4 selected devices.

To accomplish a rigorous and accurate comparison, all devices analyzed exactly the same images. Fifty inflorescence images were acquired with the Sony Xperia Z2 using the common Android camera application. Then, a simplified version of the application excluding the image capture and storage features was implemented. Basically, this version only included a simple home page with a single button for running the test. Upon test starting, the application iteratively analyzed the 50 images (stored in the device’s internal storage), registering the time taken for each one.

**Table 2:** Main relevant features of the 4 devices used for evaluating the computational efficiency of the vitisFlower application.

	Feature	Price/Release Date	Chipset	CPU	GPU	RAM Memory	Android Version
Device							
**Sony Xperia Z2**		549.0 €/2014	Qualcomm MSM8974AB Snapdragon 801	Quad-core 2.3-GHz Krait 400	Adreno 330	3 GB	5.0.1 Jelly bean
**Sony Xperia Z2 Tablet**		449.0 €/2014	Qualcomm MSM8974AB Snapdragon 801	Quad-core 2.3-GHz Krait 400	Adreno 330	3 GB	4.4.4 Kit kat
**BQ Aquaris E5**		209.90 €/2014	MediaTek MT6582	Quad-core 1.3-GHz ARM Cortex-A7	Mali-400 MP2	1 GB	4.4 Kit kat
**Motorola Moto G**		172.0 €/2013	Qualcomm MSM8226 Snapdragon 400	Quad-core 1.2-GHz Cortex-A7	Adreno 305	1 GB	4.4.2 Kit kat

The acquired set of 50 images and the benchmarking version of the vitisFlower^®^ application were used to compare its computation time running on the devices detailed in Table 2. For standardizing the state of the smartphones and minimizing the interference of other applications or services installed on them, the following testing protocol was defined and followed for carrying out the test:

1. Closing all recent applications in the device.
2. Selection of the flight mode.
3. Re-starting the device.
4. Waiting for 20 s for the operating system to completely load.
5. Starting the benchmarking version of vitisFlower.
6. Running the tests 5 times.

Once the test was finished, the application generated 5 files, including the measured computation time for each image in each of the 5 iterations. Finally, the definitive computation time for an image was calculated as the average time taken for its analysis in the 5 performed iterations.

## 3. Results and Discussion

### 3.1. Results of Performance Evaluation

**Figure 4:**
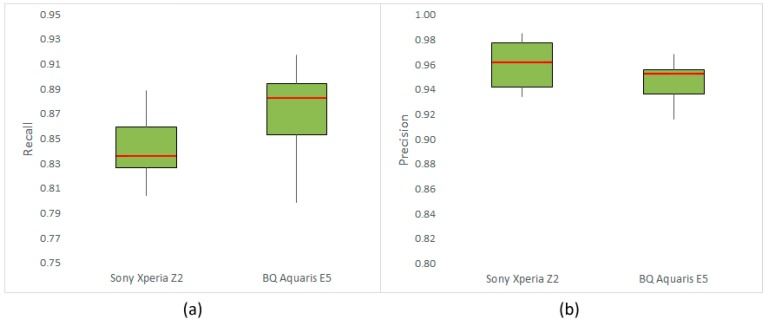
Box and whisker comparison plots for the Sony Xperia Z2 and the BQ Aquaris E5: (**a**) performance in terms of average *Recall* ($\bar{RC}$); (**b**) performance comparison in terms of average *Precision* ($\bar{PR}$).

Table 3 shows the results obtained with the 2 smartphones detailed in Table 1 in terms of average *Recall* ($\bar{RC}$) and *Precision* ($\bar{PR}$) following the testing methodology described in Section 2.4.1. Results are given in detail per variety. The overall results obtained with both devices (see Figure 4) indicated that more than 84% of flowers in the images were identified, producing less than 6% of detection errors. Furthermore, the dispersion of $\bar{RC}$ and $\bar{PR}$ values measured per variety and graphically represented in Figure 4 indicated a good stability of the application’s behavior for all of them. In this respect, only the $\bar{RC}$ value obtained by the BQ Aquaris E5 on Chenin Blanc was lower than the values achieved for other cultivars, which may be explained by non-optimum acquisition conditions and potentially by the huge degree of compactness of the flower buttons of this cultivar at this phenological stage. In this regard, the usage of the application in the field and the analysis of the acquired images led to delineating the image acquisition settings that yielded the best application behavior. These include:

- Analyzing inflorescences facing the Sun. The opposite orientation leads to light reflection and refraction patterns that can negatively affect the results.
- Casting a shadow on the inflorescence to create a homogeneous scene. If the illumination is poor due to low natural-light conditions, the use of the camera flash is recommended.

**Table 3:** Performance evaluation of vitisFlower^®^ using 2 different devices. The average *Recall* ($\bar{RC}$) and *Precision* ($\bar{PR}$) calculated from the 10 images in each grapevine variety are given.

Sony Xperia Z2			BQ Aquaris E5		
Variety	$\bar{RC}$	$\bar{PR}$	Variety	$\bar{RC}$	$\bar{PR}$
Airen	0.8223	0.9787	Merlot	0.9173	0.9517
Cabernet Sauvignon	0.8363	0.9615	Cabernet Sauvignon	0.8855	0.9531
Chardonnay	0.8770	0.9339	Chenin Blanc	0.7987	0.9563
Grenache	0.8045	0.9763	Grenache	0.8391	0.9685
Riesling	0.8411	0.9458	Riesling	0.9035	0.9212
Syrah	0.8889	0.9376	Sauvignon Blanc	0.8664	0.9557
Tempranillo	0.8308	0.9851	Semillon	0.8826	0.9158

The Sony Xperia Z2 comprises a camera sensor and a lens, which are technically more advanced than those of the BQ Aquaris E5. As can be seen in Table 1, the Xperia smartphone offers a sensor with higher image resolution, as well as a lens with a wider size and aperture. These features allow this device to produce less noise and better defined images than those captured by the BQ. Nevertheless, comparing the results obtained with both smartphones, it can be concluded that technical differences between them did not affect the application’s performance. Furthermore, the results obtained with the BQ device were slightly higher in terms of $\bar{RC}$ than those obtained with the smartphone from Sony. This outstanding result indicates that the vitisFlower^®^ application can be satisfactorily used, at least with smartphones in the mid-range, like the BQ Aquaris E5 and above. Moreover, since performance degradation has not been detected with this device, good results may be surely obtained, even with more modest smartphones.

### 3.2. Results of the Study of Computational Efficiency

**Figure 5:**
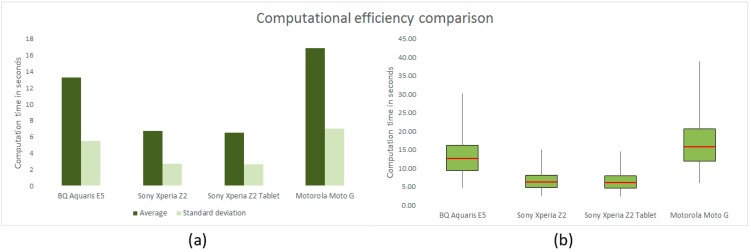
(**a**) Average and standard deviation of computation time for four different devices measured on the analysis of 50 images; (**b**) box and whisker plots for the same experiment shown in (**a**).

Figure 5 illustrates the results obtained in the experiment specified in Section 2.4.2 for evaluating the computational efficiency of the vitisFlower^®^ application running on the four devices listed in Table 2.

As expected, the Sony Xperia Z2 and Sony Xperia Z2 Tablet offered the best computational time distribution. The performance of these devices was high and virtually equal, with an average and standard deviation time of around 6.5 and 2.6 s, respectively. Since they were equipped with the same computational hardware, but different Android versions, this result reveals that the differences between both versions did not affect the application’s efficiency. On the other hand, the performance of the other two devices tested was considerably poorer. Despite this, the measured computational time for both devices (between 10 and 20 s) can be considered acceptable for allowing normal use of the application. Indeed, the BQ Aquaris E5 and Motorola Moto G (2013 version) took 13.27 and 16.83 s on average for analyzing the 50 images with a standard deviation of 5.48 and 7.01 s, respectively. Furthermore, according to the complete outcomes represented in Figure 5b by a box and whisker plot, 75% of the images were analyzed by the BQ Aquaris E5 in less than 16.24 s and by the Motorola Moto G (2013 version) in less than 20.64 s.

### 3.3. Significance of the vitisFlower^®^ Application for the Wine Industry

Flowering and fruit-set are the main determinants of grapevine yield, and fruit-set rates may be impacted by many viticultural practices, including late pruning [14], shoot tipping [26], topping [15], early defoliation [16] and spray applications of growth regulators and nutrients [19,27]. Despite its importance, limited flower counting and fruit-set estimation are currently carried out in commercial vineyards, as manual flower counting is very laborious and destructive. However, the possibility of doing it in a fast and non-destructive way, such as with the vitisFlower^®^ application, may pave the way for the early estimation of yield by the assessment of the fruit-set rates. The knowledge of this variable can help to estimate the final yield at harvest prior to bunch closure. This yield forecast can be very valuable for making decisions on vineyard management to optimize the grapevine balance between vegetative and reproductive growth and to prepare growers and wineries for the harvest operation, including scheduling and arrangements of shipping, storing, processing and trading the crop [28].

In the last few years, smart devices’ user penetration has increased exponentially worldwide, and the development of applications for a wide range of uses has grown in parallel. The user’s penetration percentage had surpassed 70% by June 2014 in many wine producing countries [29], where grape growers and farmers have adopted smartphones for their routine duties. For this reason, the development of agriculture-oriented applications, such as vitisFlower*^®^*, to provide fast and in-field, non-invasive assessment of agronomical and physiological information of the grapevines may become decision support tools for vineyard management.

## 4. Conclusions/Outlook

This paper presents an innovative smartphone Android application, called vitisFlower^®^, that provides the worldwide wine industry with a powerful tool for easily and automatically assessing flowering in the vineyard and providing useful information for yield estimation at early stages. The results of the experimentation developed in this paper demonstrate that even with modest devices, the application can be efficiently and reliably used at high rates of applicability and performance.

vitisFlower^®^ is currently freely available in Spanish, English and French via Google Play [30], being one of the first viticulture smartphone applications available worldwide The development of friendly, non-invasive applications for viticulture and other agricultural fields opens a new and profitable window for the implementation of precision agriculture strategies, aimed at optimizing the management according to the field variability.
